# The Diagnosis of Feline Leukaemia Virus (FeLV) Infection in Owned and Group-Housed Rescue Cats in Australia

**DOI:** 10.3390/v11060503

**Published:** 2019-05-31

**Authors:** Mark Westman, Jacqueline Norris, Richard Malik, Regina Hofmann-Lehmann, Andrea Harvey, Alicia McLuckie, Martine Perkins, Donna Schofield, Alan Marcus, Mike McDonald, Michael Ward, Evelyn Hall, Paul Sheehy, Margaret Hosie

**Affiliations:** 1 Sydney School of Veterinary Science, The University of Sydney, Camperdown, 2006 NSW, Australia; jacqui.norris@sydney.edu.au (J.N.); am5151@columbia.edu (A.M.); alan.marcus@sydney.edu.au (A.M.); 2 Centre for Veterinary Education, The University of Sydney, Camperdown, 2006 NSW, Australia; richard.malik@sydney.edu.au; 3 Clinical Laboratory and Centre for Clinical Studies, Vetsuisse Faculty, The University of Zurich, CH-8057 Zürich, Switzerland; rhofmann@vetclinics.uzh.ch; 4 Small Animal Specialist Hospital, 1 Richardson Place, North Ryde, Sydney, 2113 NSW, Australia; andreaharvey.cat@googlemail.com; 5 Pymble Veterinary Clinic, West Pymble, 2073 NSW, Australia; martine@thepymblevet.com.au; 6 PetCure, Glebe, 2037 NSW, Australia; donna@petcure.com.au; 7 Veterinary Diagnostic Services, The University of Glasgow, Glasgow, Scotland G61 1QH, UK; mike.mcdonald@glasgow.ac.uk; 8 Sydney School of Veterinary Science, The University of Sydney, Camden, 2570 NSW, Australia; michael.ward@sydney.edu.au (M.W.); evelyn.hall@sydney.edu.au (E.H.); paul.sheehy@sydney.edu.au (P.S.); 9 MRC—University of Glasgow Centre for Virus Research, Glasgow, Scotland G61 1QH, UK; margaret.hosie@glasgow.ac.uk

**Keywords:** veterinary science, feline leukaemia virus, FeLV diagnosis, antigen testing, PCR, cats, Australia

## Abstract

A field study was undertaken to (i) measure the prevalence of feline leukaemia virus (FeLV) exposure and FeLV infection in a cross-section of healthy Australian pet cats; and (ii) investigate the outcomes following natural FeLV exposure in two Australian rescue facilities. Group 1 (*n* = 440) consisted of healthy client-owned cats with outdoor access, predominantly from eastern Australia. Groups 2 (*n* = 38) and 3 (*n* = 51) consisted of a mixture of healthy and sick cats, group-housed in two separate rescue facilities in Sydney, Australia, tested following identification of index cases of FeLV infection in cats sourced from these facilities. Diagnostic testing for FeLV exposure/infection included p27 antigen testing using three different point-of-care FeLV kits and a laboratory-based ELISA, real-time polymerase chain reaction (qPCR) testing to detect FeLV proviral DNA in leukocytes, real-time reverse-transcription PCR (qRT-PCR) testing to detect FeLV RNA in plasma, and neutralising antibody (NAb) testing. Cats were classified as FeLV-uninfected (FeLV-unexposed and presumptively FeLV-abortive infections) or FeLV-infected (presumptively regressive and presumptively progressive infections). In Group 1, 370 FeLV-unexposed cats (370/440, 84%), 47 abortive infections (47/440, 11%), nine regressive infections (9/440, 2%), and two progressive infections (2/440, 0.5%) were identified, and 12 FeLV-uninfected cats (12/440, 3%) were unclassifiable as FeLV-unexposed or abortive infections due to insufficient samples available for NAb testing. In Groups 2 and 3, 31 FeLV-unexposed cats (31/89, 35%), eight abortive infections (8/89, 9%), 22 regressive infections (22/89; 25%), and 19 progressive infections (19/89; 21%) were discovered, and nine FeLV-uninfected cats (9/89; 10%) were unclassifiable due to insufficient samples available for NAb testing. One of the presumptively progressively-infected cats in Group 3 was likely a focal FeLV infection. Two other presumptively progressively-infected cats in Group 3 may have been classified as regressive infections with repeated testing, highlighting the difficulties associated with FeLV diagnosis when sampling cats at a single time point, even with results from a panel of FeLV tests. These results serve as a reminder to Australian veterinarians that the threat of FeLV to the general pet cat population remains high, thus vigilant FeLV testing, separate housing for FeLV-infected cats, and FeLV vaccination of at-risk cats is important, particularly in group-housed cats in shelters and rescue facilities, where outbreaks of FeLV infection can occur.

## 1. Introduction

In many countries, the prevalence of feline leukaemia virus (FeLV) infection in domestic cats has decreased since the 1970s and 1980s [1,2]. For example, in parts of Australia, Europe, and North America, prevalence rates of 1–6% have been reported in the past decade [3,4,5,6]. This decrease has been attributed to veterinary interventions, including rigorous testing combined with vaccination, and isolation or euthanasia of infected animals [2,7]. The implementation of biosecurity protocols, with or without concurrent vaccination, has effectively protected group-housed cats [2,8]. Nevertheless, studies have demonstrated that progressive FeLV infections remain common in some regions, such as Southeast Asia and Brazil, where the prevalence is 12–25% [9,10,11,12,13], and recently in Australia a prevalence of 13% (72/563) was reported, with the majority of infections identified in sick cats tested as part of routine diagnostic investigations [14]. This finding highlights that the possibility of FeLV infection should still be considered by Australian veterinarians. Indeed, the risk of FeLV infection remains sufficiently high in the general cat population that current guidelines recommend shelter staff test all incoming cats of unknown retroviral status for FeLV infection following a six-week quarantine period [8].

The pathogenesis of FeLV infection varies according to the host immune response. The main host outcomes following FeLV exposure are complete elimination of FeLV following an effective immune response (‘abortive infection’), a partially effective immune response that clears the viraemia but does not prevent proviral integration (‘regressive infection’), and persistent viraemia with proviral integration (‘progressive infection’) [2,15,16,17,18]. To differentiate between these outcomes, antigen testing (to detect viral capsid protein p27), polymerase chain reaction (PCR) testing (to detect proviral DNA), reverse-transcription (RT)-PCR testing (to detect viral RNA [vRNA]), and testing for antibodies against FeLV are required [16]. Progressively-infected cats have the poorest prognosis, with reported mortality rates of up to 90% within three years of infection, predominantly due to aplastic anaemia, lymphoma, leukaemia, or other myeloproliferative diseases [2,15,16,19,20]. Although the role of regressive FeLV infection in disease causation is not clear, an association between regressive infections and lymphoma has been observed in Australia and Canada [21,22,23]. It is important to identify FeLV infections and to differentiate regressive versus progressive infections, to guide prognosis for the individual cat and to direct clinical management.

The aims of the current study were to (i) measure the prevalence of FeLV exposure and FeLV infection in a cross-section of healthy Australian pet cats; and (ii) investigate the outcomes following natural FeLV exposure in two Australian rescue facilities.

## 2. Materials and Methods

### 2.1. Study Population

**Figure 1:**
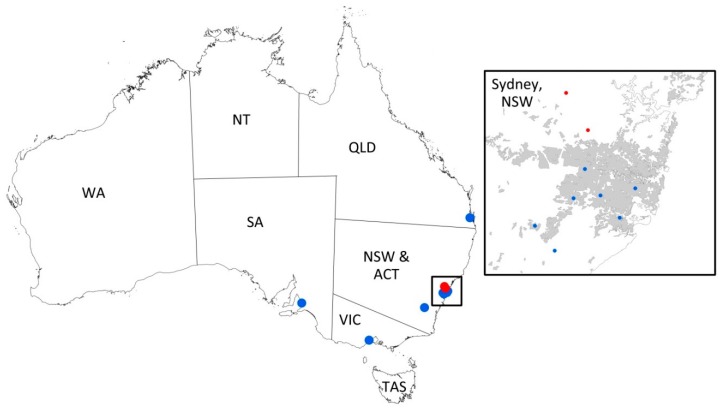
Map of Australia, showing the location of the veterinary hospitals (Group 1; blue dots) used to recruit cats from all Australian States and Territories except WA, NT, and TAS, and the location of the two rescue facilities in Sydney, NSW (Groups 2 and 3; red dots). An enlargement of Sydney and its surrounding areas is shown in the breakout box. The grey shading in the breakout box represents the ‘Built Up Areas’ of Sydney (as defined by Geosciences Australia, http://www.ga.gov.au/mapspecs/250k100k/appendixA_files/Habitation.html#Habitation Built Up Area Polygon) and is included to show the semi-rural location of the two rescue facilities and one veterinary hospital on the outskirts of Sydney. ACT = Australian Capital Territory, NSW = New South Wales, NT = Northern Territory, QLD = Queensland, SA = South Australia, TAS = Tasmania, VIC = Victoria, WA = Western Australia.

Three groups of cats in Australia, together comprising 529 cats, were included (Figure 1):

(i) Group 1 (*n* = 440) consisted of client-owned cats predominantly living in eastern Australia that were part of a case-control study investigating the effectiveness of a commercially available feline immunodeficiency virus (FIV) vaccine (Fel-O-Vax FIV^®^, Boehringer Ingelheim, Fort Dodge, IA, USA) [24]. Cats included in the current study were at least two years-of-age (according to veterinary hospital records), had outdoor access (since cats housed exclusively indoors are not exposed to FIV and FeLV unless cohabiting with an already infected cat), and had no clinical signs of illness [25]. Eleven cats that were excluded from the original study investigating the effectiveness of the FIV vaccine, as they had previous but not current outdoor exposure at the time of sampling, and did not fit the strict criteria for the case-control model, were included in the current study since they had previously been allowed outdoor access and therefore were deemed to have potentially been exposed to FeLV [24]. Complete vaccination histories were available for these cats: 145/440 (33%) had been vaccinated against FeLV, comprising 90/145 (62%) that had been vaccinated within the previous 12 months (i.e., ‘on-time’ according to the manufacturers’ guidelines) and 55/145 (38%) that had been vaccinated >12 months (‘overdue’ for vaccination). Cats had been vaccinated with one of three FeLV vaccines (36 with Fel-O-Vax Lv-K^®^, Boehringer Ingelheim, Fort Dodge, IA, USA; 108 with Fel-O-Vax F5^®^, Boehringer Ingelheim, Fort Dodge, IA, USA; one with Leucogen^®^, Virbac Animal Health, Carros, France).

(ii) Group 2 (*n* = 38) consisted of asymptomatic and sick cats group-housed in a rescue facility near Sydney, Australia. This facility was an occupied house located in a semi-rural area, with all cats allowed to roam freely during the day. Some of the more tractable cats were group-housed indoors at night. The ages of cats were recorded when available, based on their known history or estimates provided by their regular veterinarian. The age of one cat was unknown. None of the cats had been vaccinated against FIV or FeLV.

(iii) Group 3 (*n* = 51) consisted of asymptomatic and sick cats group-housed in another rescue facility near Sydney, Australia (35 km from Group 2; no cats transferred between the two facilities). This facility was an occupied house located in a semi-rural area, with all cats permanently group-housed either indoors or outdoors in large walk-through enclosures. The ages of cats were recorded when available; the ages of 41 cats were unknown. None of the cats had been vaccinated against FIV or FeLV.

Animal ethics approval for the sampling of cats in Group 1 was granted by the University of Sydney (UoS) Animal Ethics Committee (Approval number N00/1-2013/3/5920). Cats in Groups 2 and 3 were sampled and tested at the request of the facility managers following the diagnosis of progressive FeLV infections (A.H. and M.P., respectively).

### 2.2. Determination of FeLV Exposure/Infection Status

Diagnostic testing undertaken included p27 testing with three different point-of-care (PoC) FeLV antigen kits, p27 testing with a laboratory-based non-proprietary ELISA (in use since before PoC FeLV testing became available), semi-quantitative real-time proviral PCR (qPCR) testing to detect FeLV proviral DNA in leukocytes, semi-quantitative qRT-PCR testing to detect FeLV RNA in plasma, and testing for anti-FeLV neutralising antibodies (NAb). The same panel of FeLV tests was performed for all cats, on the same blood samples collected at a single time point, as outlined below. Occasionally, insufficient sample volume precluded all tests from being performed.

Whole blood samples were collected into two EDTA tubes from each cat and transported on ice to the UoS within 6 hours of collection. One tube was centrifuged for 3 min at 12,000× *g* and harvested plasma was aliquoted into two plain tubes using a sterile pipette and stored at −80 °C. At the end of the sampling period, one aliquot of frozen plasma from each cat was transported on dry ice to the University of Zurich for qRT-PCR testing and laboratory-based p27 ELISA testing, while the other aliquot of frozen plasma was thawed and transported on ice to the University of Glasgow for NAb testing. PoC testing with three FeLV kits (used concurrently) was performed with whole blood samples at the UoS, within 24 h of blood collection using the second EDTA tube, and DNA extraction from leukocytes was performed using sample collected in the same EDTA tube within 72 h of collection and stored at −80 °C for batch qPCR testing at the UoS.

Results from these analyses enabled classification of cats as FeLV-uninfected or FeLV-infected, using the following definitions (Table 1). FeLV-uninfected cats included FeLV-unexposed cats (p27-negative, qPCR-negative, NAb-negative) and FeLV-abortive infections (p27-negative, qPCR-negative, NAb-positive). FeLV-infected cats included cats with a ‘presumptively regressive infection’ (p27-negative, qPCR-positive, NAb negative or positive) and cats with a ‘presumptively progressive infection’ (p27-positive, qPCR-positive, NAb negative or positive). FeLV-vaccinated cats uncommonly produce NAb following vaccination without exposure [26,27,28], and instead, FeLV vaccination is thought to ‘prime’ the immune system and boost the NAb response following exposure [7]. Consequently, FeLV-vaccinated/FeLV-uninfected cats that tested NAb-positive were considered FeLV-vaccinated cats that had come into contact with the virus and were classified as ‘presumptively abortive infections’ (versus FeLV-unvaccinated/FeLV-uninfected cats that tested NAb-positive and were considered as ‘definitively abortive infections’). ‘Presumptively’ regressive and progressive infections were described because, in the absence of repeated testing, a transient viraemia may have been present in some cats, resulting in some regressive infections being incorrectly classified as progressive infections [1,2,29]. Detection of vRNA by qRT-PCR testing was not included in the definition for progressive infections, as has been suggested elsewhere, since residual plasma was not available from all FeLV-infected cats for testing [17,29].

#### 2.2.1. FeLV PoC p27 Testing

SNAP Combo^®^ (IDEXX Laboratories, Westbrook, ME, USA), Witness^®^ (Zoetis Animal Health, Lyon, France), and Anigen Rapid^®^ (BioNote, Gyeonggi-do, Korea) FeLV test kits were used according to the manufacturers’ instructions [14]. The specificity and sensitivity for the detection of progressive FeLV infection have been estimated to be 94% and 100% for SNAP Combo^®^, respectively, and 98% and 91% for Witness^®^ and Anigen Rapid^®^, respectively [30]. Cats were considered p27-positive if any of the three PoC kits tested returned a positive result (in combination with a positive qPCR result, otherwise a false-positive p27 result was declared), and p27-negative if all three kits returned a negative result [14].

#### 2.2.2. FeLV qPCR Testing

DNA extraction from leukocytes in EDTA-anticoagulated whole blood and semi-quantitative qPCR testing was performed to detect FeLV provirus as described previously, using a cycle threshold (C_T_) cut-off of 40 [14,31]. DNA was extracted from 200 µL of blood using a kit (QIAamp DNA Mini Kit (Qiagen, Valencia, CA, USA)), and 3 µL of DNA (approximately 100 ng) was added per 25 µL qPCR reaction [14].

qPCR analysis for genomic mammalian DNA (feline 28S rDNA gene) was also performed using a published protocol to assess the quality of the thawed DNA [14,32,33]. Samples with a C_T_ value of less than 37 were recorded as a positive result [14].

#### 2.2.3. FeLV qRT-PCR Testing

A described real-time TaqMan qRT-PCR assay was used to detect FeLV RNA in plasma (vRNA), with some modifications [31]. Briefly, total nucleic acid (TNA) was extracted from 100–200 μL of EDTA-anticoagulated plasma using the MagNA Pure LC Total Nucleic Acid Kit (Roche Diagnostics, Mannheim, Germany) and an elution volume of 100 μL. In all the extractions, negative controls consisting of phosphate buffered saline (PBS) were extracted in parallel to monitor for cross-contamination. The 25 μL qRT-PCR reaction contained 5 μL TNA, 12.5 µL 2× RT-PCR Buffer, 1 µL 25× RT-PCR Enzyme Mix (AgPath-IDTM One-Step RT-PCR Reagents, Thermo Fisher Scientific), a final concentration of 900 nM of forward primer (FeLV_U3_exo_f), 300 nM of reverse primer (FeLV_U3_exo_r), and 200 nM of fluorogenic probe (exoFeLV-U3-probe). Testing was performed using an ABI PRISM 7500 Fast Sequence Detection System (Applied Biosystems, Foster City, CA, USA). The temperature profile was 10 min at 45 °C, followed by 10 min at 95 °C, and then 40 cycles of 15 seconds at 95 °C and 45 s at 60 °C. Negative (PBS), positive (RNA standard template [31]) and extraction controls were included in each run. Not all samples were available for qRT-PCR testing.

#### 2.2.4. Laboratory-Based FeLV p27 ELISA Testing

The accuracy of laboratory-based FeLV p27 testing was compared to FeLV PoC p27 testing, with the overall PoC result being taken as correct. If a cat tested p27-negative with the laboratory-based ELISA, p27-positive with 1/3, 2/3, or 3/3 PoC test kits, and PCR-positive, it was declared truly p27-positive (and false-negative with the laboratory-based ELISA). If a cat tested p27-positive with the laboratory-based ELISA, p27-negative with 3/3 PoC test kits, and PCR-positive, it was declared p27-negative (and false-positive with the laboratory-based ELISA). Residual plasma samples from qRT-PCR testing were tested using a double-antibody sandwich ELISA using monoclonal antibodies to three epitopic regions of p27, as described [34]. The results were calculated as the percentages of a defined positive control (plasma from an experimentally FeLV-infected specific pathogen free cat) run with each test; samples that tested >4% of the positive control were considered p27-positive [35]. Not all samples were available for p27 antigen testing by laboratory-based ELISA.

#### 2.2.5. FeLV NAb Testing

NAb were detected using the focus reduction test described [26]. Briefly, QN10 cells were incubated overnight in 12 well plates (5 × 10^4^ cells/mL) before the addition of 4 × 10^2^ FFU/mL of FeLV-A/Glasgow-1 that had been incubated for 2 hours with an equal volume of two-fold serial dilutions of plasma samples (1/4, 1/8, 1/16, and 1/32) and the residual infectivity was measured. The NAb titres were recorded as the reciprocals of the plasma dilutions that reduced the focus count of FeLV by 75% compared with the virus control that had been incubated without plasma. A NAb titre of 4 was considered weakly NAb-positive, while a titre of 32 was considered strongly NAb-positive. Some samples had insufficient volume for NAb testing.

### 2.3. Statistical Analysis Including Mapping of Cases of FeLV Exposure and Infection

Numerical analyses were performed using the statistical software Genstat 16th Edition (GenStat 16th Edition for Windows, VSN International, Hemel Hempstead, United Kingdom) and R (The R Foundation, Vienna, Austria). A Shapiro–Wilk test was used to assess data for normality. Normally distributed data were analysed using restricted maximum likelihood (REML) modelling, while non-normal data were analysed using Mann–Whitney *U*-tests. Since C_T_ values were not normally distributed, both median and geometric mean values are reported. Two-tailed Fisher’s exact tests were applied to compare binomial outcomes, except when the expected frequency was ≥5 when a Chi-squared test was used. Cohen’s Kappa Index Value (κ) was calculated to assess the level of agreement between the laboratory-based p27 ELISA and p27 PoC testing, using the standard formula (κ = 1 − (1 − Po)/(1 − Pe)), where Po was the observed agreement and Pe was the expected agreement (0.5). Cases were mapped based on reported postcode of residence by joining these to a postcode shapefile of Australia (ArcGIS version 10.1 [ESRI, Redlands, CA, USA]). Potential geographic ‘hot spots’ of FeLV exposure (i.e., abortive infections) and FeLV infection (i.e., regressive and progressive infections) in Group 1, based on postcode longitude and latitude centroid coordinates, were investigated using the scan statistic SaTScan version 9.4.1 (Boston, MA, USA, www.satscan.org). A Bernoulli model (case-control) was used, and both circular and elliptical scanning windows of up to 50% of the population at risk were investigated to identify clusters based on likelihood statistics (999 Monte Carlo simulations).

## 3. Results

### 3.1. Description of Study Population (n = 529)

Cats in Group 1 (*n* = 440) were aged 2–20 years (median 7 years; interquartile range [IQR] 5–10 years), comprising 229 (52%) castrated males, 207 (47%) spayed females, and four (1%) entire males. The majority were non-pedigree cats (371/440, 84%).

Cats in Group 2 (*n* = 38) were aged 1–14 years (median 4 years; IQR 3–10 years), comprising 15 (39%) castrated males and 23 (61%) spayed females. Most were non-pedigree cats (36/38, 95%).

Cats in Group 3 (*n* = 51) were aged 3 months to 5 years (median 1 year; IQR 0.4–2 years), comprising 23 (45%) castrated males, 27 (53%) spayed females, and one entire female (2%). Most were non-pedigree cats (49/51, 96%).

The median ages of Groups 1, 2, and 3 were significantly different (7 years, 4 years, and 1 year; *p* < 0.01; REML testing). The proportion of males and females in each group was similar (*p* = 0.11; REML testing).

### 3.2. Identification of FeLV-Infected Cats

In total, 52 FeLV-infected cats, including 31 presumptively regressive infections (Group 1—9/440, 2%; Group 2—7/38, 18%; Group 3—15/51, 29%) and 21 presumptively progressive infections (Group 1—2/440, 0.5%; Group 2—7/38, 18%; Group 3—12/51, 24%), were identified (Table 2).

Considering results from all three groups together, the median age of presumptively regressively-infected cats (range 2–16 years, median 10 years) was greater than the median age of presumptively progressively-infected cats (range 0.4–11 years, median 5 years), although the difference did not reach statistical significance (*p* = 0.064; Mann–Whitney *U*-test), and the ages of only 3/27 FeLV-infected cats in Group 3 were known. There was no sex predilection for either presumptively regressive or progressive infections (*p* = 0.71 and *p* = 0.38, respectively; Fisher’s exact tests), nor any breed predilection (i.e., non-pedigree versus pedigree cats; *p* = 1.00 for regressive infections, *p* = 0.10 for progressive infections; Fisher’s exact tests).

In Group 1, three presumptively regressively-infected cats and one presumptively progressively-infected cat had been vaccinated against FeLV, although these four cats had not been FeLV tested prior to initial vaccination.

### 3.3. Detection of FeLV Provirus by qPCR

**Figure 2:**
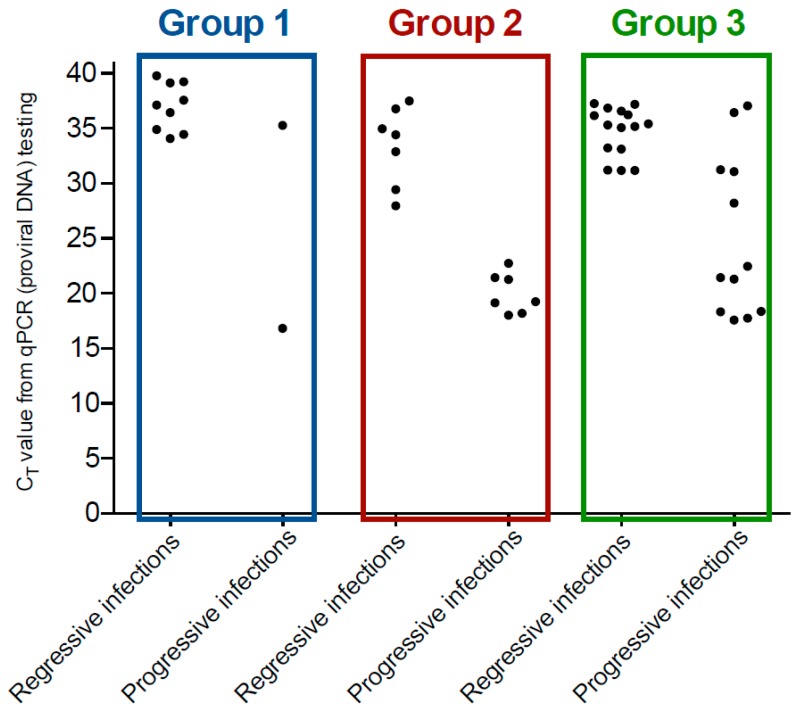
Scatter plot of C_T_ values (*y*-axis) from FeLV qPCR testing of EDTA-anticoagulated whole blood samples for the three groups of cats by type of FeLV infection (*x*-axis). Samples with a C_T_ value of less than 40 were recorded as a positive result. Presumptively progressively FeLV-infected cats (*n* = 21) recorded a significantly lower median qPCR C_T_ value (i.e., higher proviral load) compared to presumptively regressively FeLV-infected cats (*n* = 31) (*p* < 0.001).

EDTA-anticoagulated samples were available from all 529 cats for qPCR testing. Presumptively progressively-infected cats demonstrated significantly lower median qPCR C_T_ values (median 21, geometric mean 23, range 17–37, IQR 18–29) compared to presumptively regressively-infected cats (median 35, geometric mean 35, range 28–40, IQR 33–37; *p* < 0.001; Mann–Whitney *U*-test). There was some overlap of qPCR C_T_ values between presumptively regressively and progressively-infected cats (Figure 2) and five presumptively progressively-infected cats (one in Group 1 and four in Group 3) had low proviral loads (C_T_ values > 30).

The median C_T_ value from feline 28S rDNA qPCR testing was 29 (geometric mean 29, range 27–36, IQR 28–30). All samples, with one exception, returned a C_T_ value below 35, confirming the adequate quality of the thawed DNA. The one exception (C_T_ value of 36.4) was from a cat in Group 1 that tested p27-negative and qPCR-negative for FeLV proviral DNA.

### 3.4. Detection of FeLV RNA by qRT-PCR

**Figure 3:**
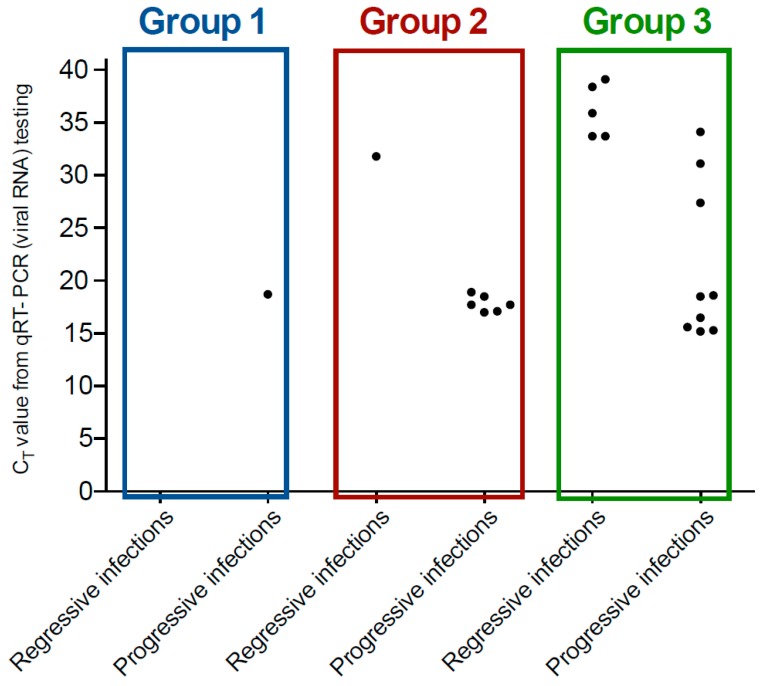
Scatter plot of C_T_ values (*y*-axis) from FeLV qRT-PCR testing of EDTA-anticoagulated plasma samples for the three groups of cats by type of FeLV infection (*x*-axis). Samples with a C_T_ value of less than 40 were recorded as a positive result. Presumptively progressively FeLV-infected cats (*n* = 18) were significantly more likely to test vRNA-positive than presumptively regressively FeLV-infected cats (*n* = 28; *p* < 0.001), and when presumptively progressively-infected cats tested vRNA-positive they recorded a significantly lower median qRT-PCR C_T_ value than presumptively regressively-infected cats (*p* < 0.001). Samples from FeLV-infected cats that tested vRNA-negative are not shown (22 presumptively regressively-infected cats and 2 presumptively progressively-infected cats; refer to Table 4 for more details).

Insufficient plasma was available from three presumptively regressively-infected and three presumptively progressively-infected cats to perform qRT-PCR testing for FeLV RNA (Table 3). Presumptively progressively-infected cats were more likely to test vRNA-positive than presumptively regressively-infected cats (16/18, 89% versus 6/28, 21%; *p* < 0.001; Chi-squared test; Figure 3). When presumptively progressively-infected cats tested vRNA-positive, they recorded significantly lower median qRT-PCR C_T_ values (median 18, geometric mean 19, range 15–34, IQR 17–19) than presumptively regressively-infected cats (median 35, geometric mean 35, range 32–39, IQR 34–38; *p* < 0.001; Mann–Whitney *U*-test; Figure 3). The two presumptively progressively-infected cats testing vRNA-negative tested p27-positive using only one PoC kit (SNAP Combo^®^; Table 4). Presumptively regressively-infected cats that tested vRNA-positive had significantly lower median qPCR C_T_ values (i.e., higher levels of proviral DNA) than presumptively regressively-infected cats that tested vRNA-negative (*p* = 0.008; Mann–Whitney *U*-test). The same was true for presumptively progressively-infected cats (*p* < 0.001; Mann–Whitney *U*-test).

None of the 39 samples available from FeLV-uninfected cats tested vRNA-positive.

### 3.5. Laboratory-Based FeLV p27 ELISA

Insufficient plasma was available from seven presumptively regressively-infected and four presumptively progressively-infected cats for testing with the laboratory-based FeLV p27 ELISA. Of the remaining 41 FeLV-infected cats, 0/24 presumptively regressively-infected and 15/17 presumptively progressively-infected cats tested p27-positive. None of the 38 samples available from FeLV-uninfected cats tested p27-positive. There was 98.7% test agreement (78/79) between testing with the laboratory-based ELISA and PoC testing with SNAP Combo^®^ (κ = 0.962, 95% confidence interval [CI] 0.887-1), 98.7% test agreement (78/79) between ELISA and Witness^®^ (κ = 0.960, 95% CI 0.882-1), and 97.5% test agreement (77/79) between ELISA and Anigen Rapid^®^ (κ = 0.918, 95% CI 0.806-1).

### 3.6. Detection of FeLV NAb

Insufficient plasma was available for NAb testing from 12 FeLV-uninfected cats (10 FeLV-vaccinated and 2 FeLV-unvaccinated) in Group 1, nine FeLV-uninfected rescue cats, two presumptively regressively-infected cats, and six presumptively progressively-infected cats (Table 3).

**Figure 4:**
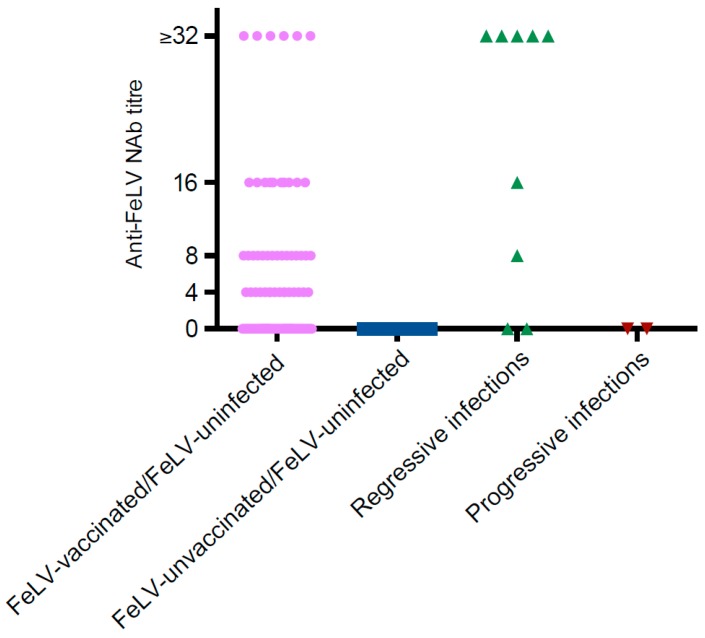
Summary of anti-FeLV NAb results in Group 1 (*n* = 428), recorded as the reciprocals of the plasma dilutions that reduced the focus count of FeLV by 75% compared with the virus control that had been incubated without plasma. A NAb titre of 4 was considered weakly NAb-positive, while a titre of 32 was considered strongly NAb-positive. Sufficient sample was available from 131 FeLV-vaccinated/FeLV-uninfected cats, 286 FeLV-unvaccinated/FeLV-uninfected cats, 9 presumptively regressively FeLV-infected cats, and 2 presumptively progressively FeLV-infected cats for NAb testing.

Considering FeLV-uninfected cats in Group 1, there were 370 NAb-negative results (i.e., FeLV-unexposed cats; 370/440, 84%), and 47 NAb-positive results (47/440, 11%; Table 3 and Figure 4). Of the 370 FeLV-unexposed cats, 84 cats were FeLV-vaccinated (including 42 vaccinated on-time and 42 overdue for vaccination) and 286 cats were FeLV-unvaccinated. Of the 47 NAb-positive cats, all were FeLV-vaccinated (17 with Fel-O-Vax Lv-K^®^, 30 with Fel-O-Vax F5^®^) and were therefore considered presumptively abortive infections (i.e., vaccinated and exposed). Most of these cats (37/47, 79%) had been vaccinated within 12 months of testing, with the remainder overdue for vaccination by between 3 days and 5.6 years. There was a strong association between FeLV vaccination and the presence of NAb (*p* < 0.001; Chi-squared test).

Considering FeLV-uninfected cats from the two rescue facilities together, 31/89 (35%) tested NAb-negative and were classified as FeLV-unexposed (Group 2—15/38, 39%; Group 3—16/51, 31%). Four FeLV-uninfected cats in Group 2 (4/38, 11%) and four in Group 3 (4/51, 8%) tested NAb-positive and were classified as definitively abortive infections.

Considering FeLV-infected cats from all three groups together, presumptively regressively-infected cats were more likely than presumptively progressively-infected cats to test NAb-positive (27/29, 93% versus 3/15, 20%; *p* = 0.000002; Fisher’s exact test). Three of the 27 NAb-positive samples from presumptively regressively-infected cats were from FeLV-vaccinated individuals (two on-time, one overdue; Table 4). The two presumptively regressively-infected cats that tested as NAb-negative were FeLV-unvaccinated. The one presumptively progressive infection that had been vaccinated on-time against FeLV was NAb-negative, while the three presumptively progressively-infected cats that tested NAb-positive were FeLV-unvaccinated (Table 4).

### 3.7. FIV Infection Status and FeLV/FIV Co-Infections

Thirty-nine cats were identified as FIV-infected based on FIV PoC testing [25], comprising 32 cats from Group 1 (32/440, 7%), three cats from Group 2 (3/38, 8%), and four cats from Group 3 (4/51, 8%). FIV-infected cats were aged 3–16 years (median 7 years; IQR 5–9 years), comprising 27 (69%) castrated males, 11 (28%) spayed females, and one (3%) entire male. Most FIV-infected cats were non-pedigree cats (35/39, 90%). Male cats were significantly more likely to be FIV-infected than female cats (*p* = 0.008; Fisher’s exact test; odds ratio 2.6, 95% CI 1.3–5.3). The age of only one FIV-infected cat in Group 3 was known.

Five cats infected with FeLV, two presumptively regressively-infected and three presumptively progressively-infected, were co-infected with FIV. FeLV/FIV co-infected cats were as likely to be presumptively progressively-infected as presumptively regressively-infected, compared to cats infected with only FeLV (3 and 2 versus 18 and 29, respectively; *p* = 0.64; Fisher’s exact test). Cats were more likely to be infected with just one retrovirus (either FeLV or FIV) than to be co-infected (*p* < 0.001; Chi-squared test). Considering only FeLV-uninfected cats, FIV-infected cats were as likely to test NAb-positive as FIV-uninfected cats (2/31 versus 43/386; *p* = 0.56; Fisher’s exact test).

### 3.8. Mapping of Cases of FeLV Exposure and Infection (Group 1 Only)

**Figure 5:**
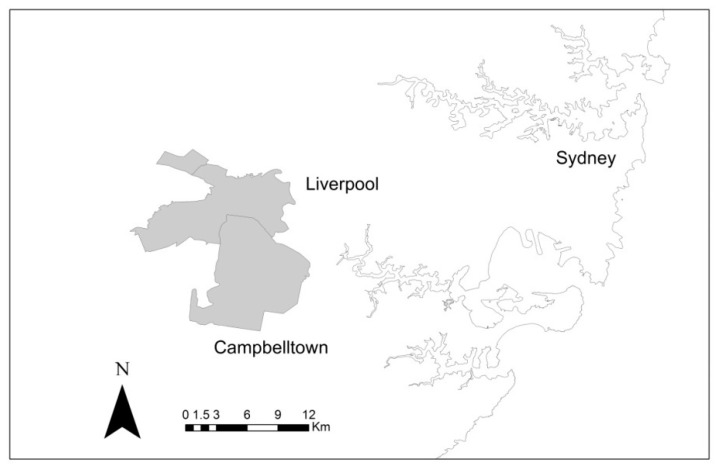
Scanning windows were used to investigate the possibility of clusters of FeLV exposure/infection in Group 1. Based on a scanning window of up to 10% of the population at risk, a cluster of abortive infections (i.e., FeLV-exposed cats) was identified in western Sydney, New South Wales (postcodes 2170, 2173, and 2177; grey shading).

Using circular and elliptical scanning windows, a cluster of abortive infections (i.e., FeLV-exposed cats) was consistently identified in western Sydney, New South Wales (NSW; Figure 5). Based on an elliptical scanning window of up to 10% of the population at risk, this cluster included postcodes 2170, 2173, and 2177, encompassing an area between Liverpool and Campbelltown (latitude 33.934° S, longitude 150.912° E). Within this cluster there were 15 abortive infections out of a sample population of 40 cats. Based on this scanning window, 4.5 cases were expected, an observed-to-expected ratio of 3.34 (*p* = 0.0084).

Also using circular and elliptical scanning windows, two potential geographical ‘hot spots’ of FeLV infection were identified in Group 1, one in western Sydney, NSW (postcode 2145; 4 cases reported, 1.6 cases expected), and one in Geelong, Victoria (postcodes 3218, 3219 and 3220; 3 cases reported, 0.58 cases expected). However, neither were statistically significant (*p* ≥ 0.60).

### 3.9. Follow-up of Rescue Cats and FeLV-Infected Cats (Groups 1 and 2 Only)

All FeLV-uninfected cats in Groups 2 and 3 were vaccinated against FeLV with Fel-O-Vax F5^®^ or Leucogen^®^ (two primary vaccinations given one month apart, as per the manufacturers’ instructions). Husbandry advice and assistance with FeLV/FIV testing of new animals were also provided to both rescue facilities to reduce the incidence and impact of retroviral disease in the cat populations. Cats in Group 2 were closely followed clinically and sampled regularly; repeated testing of presumptively progressively-infected cats (tested up to six times over two years) confirmed all seven remained p27-positive and were likely progressively-infected according to the European Pharmacopoeia (p27-positive for three consecutive weeks or on five occasions between weeks 3 and 15 post-exposure [36]). A summary of outcomes for presumptively regressively and progressively-infected cats in Groups 1 and 2 is provided in Table 5; at least 14 were deceased at the time of writing, including 6/16 regressively-infected cats and 8/9 progressively-infected cats, and 7/16 regressively-infected cats were lost to follow-up. Cats in Group 3 were lost to follow-up.

## 4. Discussion

This study reports a comprehensive analysis of the prevalence of FeLV exposure and FeLV infection of cats in Australia. Results from Group 1 demonstrated that, despite the relatively low overall prevalence of FeLV infection in the general pet population, a finite risk of exposure to FeLV is present, with 47 FeLV-uninfected cats (11%) in the current study testing positive for NAb to FeLV. Presumptively regressive infections were 4.5 times more common than presumptively progressive infections in this cohort, higher than other ratios reported in the literature (0.7–1.5) [18,33,35,37,38], including the only other Australian study to use p27 detection and PCR testing for proviral DNA [37]. The differences in ratios may in part reflect differences in recruitment criteria between the various studies, for example, the large proportion of healthy cats recruited in our Group 1 compared to other surveys that included a variable proportion of ‘sick cats’. In addition, the cohort for the other Australian study was recruited from a single city (Sydney), whereas the current study recruited cats from a wider area [37]. The prevalence of progressive infections in the healthy cat population in Australia appears to have remained stable over the past 20 years at 0–2% [5,39,40]. Samples from Western Australia were not available for testing as part of Group 1, which was unfortunate as its capital, Perth, is thought to have the highest FeLV prevalence of any Australian state or territory [5].

Results from two rescue facilities experiencing symptomatic FeLV outbreaks (Groups 2 and 3) demonstrated that clusters of cases occur in Australia in exposed, immunologically naïve cats, with a mixture of abortive, regressive, and progressive infections identified. The ratio of presumptively regressive:progressive infections in these cohorts was 1–1.25, suggesting that progressive infections are more likely in multi-cat situations compared to the general pet cat population [38], probably as a consequence of a combination of factors including higher virus exposure, higher infective doses, and higher stress levels resulting from the proximity to numerous other cats [41]. With time, and removal of progressively-infected cats due to death, illness, and/or euthanasia, the regressive to progressive ratio in Groups 2 and 3 may have increased. The lower median ages of cats in Groups 2 and 3 compared to Group 1 also likely contributed to the higher rates of progressive infections observed, as age-related immunity to FeLV develops in older cats [42]. Despite the large number of viraemic FeLV-infected cats at both rescue facilities, 39% (15/38) and 31% (16/51) of cats, respectively, were FeLV-unexposed (i.e., FeLV-uninfected and NAb-negative). This perhaps reflects the solitary nature of many cats even when group-housed [43], although abortive infections with primarily a cell-mediated rather than a humoral immune response (resulting in a NAb-negative result) are also a possibility (as evidenced by the two presumptively regressively-infected cats in Group 1 that tested NAb-negative) [7,18]. It is also possible that cats in Groups 2 and 3 were tested relatively early during outbreaks of FeLV infection at the two facilities, and that with repeated testing at later time points more cats might have demonstrated exposure to FeLV. Moreover, it is possible that a minimal humoral immune response after FeLV exposure could have been detectable in some of these cats using a recently described alternative approach to detect antibodies directed against the FeLV transmembrane antigen p15E [44]. In response to these outbreaks, we recommend that all young cats in Australia in contact with cats of unknown retroviral status should have their FeLV status determined by PoC testing and, if negative, be vaccinated against FeLV to protect them against the development of progressive infection [1,2]. Given that no benefit has been demonstrated for vaccinating cats with regressive infections against FeLV, and considering the risk of feline injection site sarcomas associated with FeLV vaccination [45], it is ideal to perform both FeLV PoC and PCR testing prior to vaccination. Currently, it is estimated only 2% of Australian client-owned cats are vaccinated against FeLV (Phillip McDonagh per comms, Technical Service Manager, Boehringer Ingelheim Animal Health, Australia). In addition, shelters and boarding facilities that allow contact between cats should perform FeLV testing of all incoming cats during a six-week quarantine period (followed by FeLV vaccination of FeLV-uninfected cats prior to co-mingling), with segregated housing for all FeLV-infected cats [8].

Presumptively regressively-infected cats were more likely to test NAb-positive than presumptively progressively-infected cats, highlighting the role an individual cat’s humoral immune response plays in determining the outcome of FeLV exposure. Despite vaccination against FeLV, and irrespective of which type of FeLV vaccine is administered, regressive infections still occur in some vaccinated cats following natural or experimental challenge with FeLV [17,27,46,47,48,49]. In the current study, it was impossible to determine whether the three presumptively regressively-infected cats that had been vaccinated against FeLV had been infected prior to initial vaccination, since no pre-vaccination FeLV testing had been performed. The impact of regressive FeLV infections on the health of cats is largely unknown, as few studies have monitored cohorts of regressively-infected cats longitudinally under field conditions to determine long-term outcomes. Some authors have suggested that regressive FeLV-infected cats have a similar life-expectancy to cats unexposed to FeLV and that FeLV-associated disease is unlikely to develop [2,7]. Conversely, reactivation of regressive FeLV infection constrained in the bone marrow has been demonstrated experimentally, with and without the administration of corticosteroids [17,20,50], and care is suggested when treating regressively FeLV-infected cats with immunosuppressive medications (e.g., chemotherapy, chlorambucil, ciclosporin) [16,47]. Further investigations on the impact of regressive FeLV infections on feline health under field conditions is warranted. Identification of regressively-infected cats (such as the 31 cats in the current study) and age-matched control cats will greatly facilitate longitudinal case-control studies.

Six presumptively regressively-infected cats (6/28, 21%) tested vRNA-positive, consistent with previous observations that some regressively-infected cats are transcriptionally active (i.e., producing vRNA), despite testing p27-negative and virus isolation negative [17,28]. Based on the ability of regressively-infected cats (as well as progressively-infected cats) to transmit FeLV to other cats via blood transfusion [51,52], this detection of vRNA indicates that there is a risk of FeLV transmission from regressively-infected cats to naïve cats in the field via cat bite injuries and the possible transfer of minute amounts of blood, particularly if the aggressor cat has significant periodontal disease. It has also been suggested that transcriptionally active, regressively-infected cats are at greater risk for reactivation of active FeLV infection than regressively-infected cats that test vRNA-negative [29]. It is possible that repeated qRT-PCR testing may have shown that more regressively FeLV-infected cats in the study were transcriptionally active, since repeated qRT-PCR testing is sometimes necessary to diagnose such cases, reflecting fluctuations in vRNA levels in blood [17].

It could be speculated that the 47 FeLV-uninfected cats in Group 1 that tested NAb-positive and were classified as abortive infections had never been exposed to FeLV, and only tested NAb-positive in response to FeLV vaccination. However, we considered this scenario unlikely, based on results from other studies. Firstly, a laboratory-based study of cats vaccinated with Fel-O-Vax Lv-K IV^®^ (distributed as Fel-O-Vax F5^®^ in Australia), and monitored for production of NAb using the same NAb assay as used in the current study, reported that 0/10 cats produced NAb prior to FeLV challenge [27]. Another laboratory-based study of cats vaccinated with Fel-O-Vax Lv-K^®^, and using a NAb assay based on infection of AH9327 cells with vaccine-homologous virus (FeLV-A/61E), found 0/8 cats were NAb-positive prior to FeLV challenge [28]. In view of these results, we felt it appropriate to classify these 47 FeLV-vaccinated cats (47/440; 11%) as presumptively abortive infections, especially considering that two cats tested strongly NAb-positive and neither had been vaccinated for over six years. The possibility of a cluster of FeLV exposure in western and southwestern Sydney further increased our suspicion that this result represented a true estimate of the prevalence of abortive infections in Group 1. Further work, however, to investigate whether FeLV-vaccinated cats in the field can produce NAb without natural exposure, would be worthwhile. The frequency of abortive infections in the field is not well documented, with a study from Southern Germany identifying such infections using an anti-p45 antibody ELISA (p45 being the non-glycosylated form of gp70, the FeLV surface envelope glycoprotein) at a prevalence of 4% (22/495), in a population with 1% regressive infections (6/495) and 2% progressive infections (9/495) [18]. Abortive infections have been reported to occur in 20–30% of cats challenged with FeLV under laboratory conditions, although this rate varies depending on the age of exposed cats, route of infection and challenge dose [2,16]. It is even possible that the rate of abortive infections in the current study was underestimated, since the NAb assay might not have detected abortive infections in cats with predominantly cell-mediated immune responses.

A limitation of the current study was that cats were only available for testing at a single time point (with the exception of FeLV-infected cats in Group 2), so that we classified cats tentatively as ‘presumptively regressive and progressive infections’ according to their p27, qPCR, and NAb results, irrespective of their qRT-PCR result. In the absence of repeated testing, this may have meant that some cats were misclassified. For example, one cat in Group 3 (#12) that tested p27-positive with only 1/3 PoC test kits (SNAP Combo^®^), was p27-negative with the laboratory-based ELISA, had a qPCR C_T_ value of 36.4, tested vRNA-negative, and was strongly NAb-positive, was categorised as a presumptively progressive infection (Table 4). The high qPCR C_T_ value (low proviral load) of this cat, in combination with an absence of vRNA, 3/4 p27-negative test results, and the presence of NAb, were otherwise suggestive of a regressive infection with the absence of significant antigenemia. This cat demonstrated the difficulties with discordant test result interpretation and of categorising cats into the different FeLV infection outcomes, particularly when data is only available from a single time point. The potential for errors with sample handling and labelling or laboratory contamination leading to discordant and unexpected results is unlikely, but cannot be absolutely excluded. Regardless of the classification system employed, p27-positive cats had mostly low qPCR C_T_ values (15/21 less than 25), while p27-negative cats had mostly high qPCR C_T_ values (29/31 higher than 30) [6,33]. As anticipated, presumptively progressively-infected cats were found to be more likely vRNA-positive than presumptively regressively-infected cats [29]. Another cat in Group 3 (#50) categorised as a presumptively progressive infection might also have had an early regressive infection, since it tested p27-negative with the laboratory-based ELISA and was strongly NAb-positive, demonstrating an effective immune response that is usually found only in cats with regressive infections [7,27,28]. The third presumptively progressive infection that tested strongly NAb-positive in Group 3 (#3) displayed discordant results, having a strongly p27-positive result with the laboratory-based ELISA (43.5%), but was only p27-positive with 1/3 PoC test kits (SNAP Combo^®^), had a very low proviral load (qPCR C_T_ value of 37.1), and was vRNA-negative. A cat with such a high p27 antigen result would typically display a higher proviral load and test vRNA-positive. Additional plasma testing at the University of Zurich (results not shown) confirmed that this cat tested qRT-PCR-negative using three additional sets of primers (including U3, env, and gag [35]). While prolonged storage and repeated freeze–thaw cycles may have contributed to the negative vRNA result and also, to some extent, the low provirus load, an alternative explanation could be the presence of a focal infection in this cat, with sequestered virus replication in the absence of virus replication in the peripheral blood. Focal FeLV infections were first described in the pre-PCR era, when about 10% of blood samples that tested positive with a p27 laboratory-based ELISA (detection of soluble antigen) were negative by virus isolation [53]. Testing of some of these ’discordant’ samples at a later date with a nested PCR found a proportion of these samples were PCR-positive (13/39, 33%) [54]. We would expect that cats with a focal infection would also test vRNA-negative. To the best of our knowledge, cat #3 in Group 3 is the first suspected case of a focal infection that has appeared in the literature in over 20 years. It would be useful to follow this cat over time to observe whether it succumbs to FeLV-related disease, and whether PCR testing of different tissue types at post-mortem could identify the location/s of the suspected focal infection. These three cats in Group 3 (#12, #50, and #3), and two additional cats in Group 3 with low proviral loads (qPCR C_T_ >30) but positive p27 test results (#9 and #40), all originated from a rescue facility with known ongoing natural FeLV exposure; only one cat in the case-control study (Group 1, cat #330), showed a similar pattern with varying p27 results (positive with 1/3 PoC test kits) and a low proviral load (qPCR C_T_ value of 35.3). Our results highlight the complexities associated with FeLV diagnosis, particularly in environments with acute infections, in which some cats are in the initial phase of FeLV infection and when the host–virus equilibrium has not yet been established [17,35,47]. Our results also highlight the need for considering a range of diagnostic testing methodologies and for repeated testing to accurately determine an individual cat’s FeLV infection status, a process which can be difficult in clinical practice.

## 5. Conclusions

Despite the reported decrease in the prevalence of FeLV infection in pet cats living in Europe, Australia, and the USA, the threat of regressive and progressive FeLV infections persists, with regressive infections being more than four times as common as progressive infections in pet cats in Australia. The extent of FeLV exposure in the owned feline population and the association between FeLV infection and various diseases (e.g., lymphoma, cytopenias) is generally underestimated; qPCR testing for FeLV provirus, qRT-PCR testing for vRNA, and testing for anti-FeLV NAb is not commonly performed in clinical practice or population surveys. Understanding the role of regressive infections in FeLV-associated disease and transmission, specifically in relation to the development of lymphoma, and particularly in cats in which the virus is transcriptionally active, will require further field investigations. It is notable that the prevalence of FeLV exposure in pet cats was more than four times greater than the prevalence of FeLV infection in our study, suggesting a higher potential risk than previously documented in Australia. Consequently, veterinarians should consider vaccinating any FeLV-uninfected cat with outdoor access against FeLV, as well as any cats that are group-housed with cats of unknown retroviral status, to reduce the risk of progressive FeLV infection developing, especially in the first few years of a cat’s life. Rescue facilities that allow group-housing should be especially vigilant about screening new arrivals for FeLV during an initial quarantine period, keeping cats in isolation before co-mingling of animals is permitted. When investigating FeLV infection, utilisation of a panel of different tests, and repeated testing, may be required to accurately categorise the infection as regressive, progressive, or focal.

## Figures and Tables

**Table 1:** Definitions used for defining each cat’s FeLV status in the current study, based on results from the panel of FeLV tests performed.

FeLV Infection Status	Category	Results from FeLV Tests Performed		
		PoC Testing (No. of Positive Kits)	qPCR	Nab ^1^
**FeLV-unexposed**		0/3	−	−
**FeLV-uninfected**	Definitively abortive infection (FeLV-unvaccinated cats)	0/3	−	+
	Presumptively abortive infection (FeLV-vaccinated cats)	0/3	−	+
**FeLV-infected**	Presumptively regressive infection	0/3	+	−/+
	Presumptively progressive infection	1/3, 2/3 or 3/3	+	−/+

+ = positive result, − = negative result. ^1^ There was insufficient sample volume from some cats for NAb testing.

**Table 2:** Summary of results from the current study.

Group	Description	FeLV-Uninfected ^1^	FeLV-Infected		
			Presumptively Regressive Infections ^2^	Presumptively Progressive Infections ^3^	Ratio Regressive:Progressive Infections
Group 1 (*n* = 440)	Healthy, client-owned cats recruited predominantly from eastern Australia as part of a study into the effectiveness of a commercially available FIV vaccine	429/440 (98%), including 47/440 (11%) presumptively abortive infections	9/440 (2%), including 0/8 with detectable circulating vRNA	2/440 (0.5%)	4.5
Group 2 (*n* = 38)	Mixture of healthy and sick cats being group-housed at a rescue facility with recent cases of FeLV infection	24/38 (63%), including 4/38 (11%) abortive infections	7/38 (18%), including 1/6 (17%) with detectable circulating vRNA	7/38 (18%)	1.0
Group 3 (*n* = 51)	Mixture of healthy and sick cats being group-housed at a rescue facility with recent cases of FeLV infection	24/51 (47%), including 4/51 (8%) abortive infections	15/51 (29%) including 5/14 (36%) with detectable circulating vRNA	12/51 (24%)	1.25

^1^ FeLV-uninfected cats were defined as those that were p27-negative with three PoC test kits and proviral qPCR-negative. ^2^ FeLV-infected cats with presumptively regressive infections were defined as those that were p27-negative with three PoC test kits and proviral qPCR-positive. ^3^ FeLV-infected cats with presumptively progressive infections were defined as those that were p27-positive with at least one of three PoC test kits and proviral qPCR-positive.

**Table 3:** Summary of results from NAb testing, laboratory-based p27 testing, and qRT-PCR testing. Not all samples were available for testing. NP = not performed.

Group		Negative NAb Result (<4)	Positive NAb Result					Positive Laboratory-Based p27 ELISA	Positive qRT-PCR
			4 (weak)	8	16	≥32 (strong)	Total NAb Positive		
1	FeLV-uninfected (*n* = 429)	370	16	15	10	6	47/417 ^1^	NP	NP
1	Presumptively regressive infections (*n* = 9)	2	0	1	1	5	7/9	0/6	0/8
1	Presumptively progressive infections (*n* = 2)	2	0	0	0	0	0/2	1/1	1/1
2	FeLV-uninfected (*n* = 24)	15	0	0	2	2	4/19	0/18	0/19
2	Presumptively regressive infections (*n* = 7)	0	0	1	0	5	6/6	0/5	1/6
2	Presumptively progressive infections (*n* = 7)	1	0	0	0	0	0/1	6/6	6/6
3	FeLV-uninfected (*n* = 24)	16	0	1	1	2	4/20	0/20	0/20
3	Presumptively regressive infections (*n* = 15)	0	0	0	1	13	14/14	0/13	5/14
3	Presumptively progressive infections (*n* = 12)	9	0	0	0	3	3/12	8/10	9/11

^1^ 10/12 samples not available for NAb testing were from FeLV-vaccinated cats.

**Table 4:** Summary of individual results for presumptively regressively-infected (*n* = 31) and presumptively progressively-infected cats (*n* = 21), arranged in descending order of C_T_ value for qPCR testing (i.e., from lower levels of FeLV provirus to higher levels of FeLV provirus). Occasionally, there was insufficient sample volume for laboratory-based p27 ELISA, qRT-PCR, and NAb testing. + = positive result, − = negative result, NP = not performed.

Cat ID	Presumptive Category of Infection	FeLV Vaccination Status	Test Performed				
			PoC Testing (No. of Positive Kits)	Laboratory-Based p27 ELISA	qPCR C_T_	qRT-PCR C_T_	NAb Assay
Group 1 - #41	Regressive	Unvaccinated	0/3	-	39.8	-	-
Group 1 - #386	Regressive	Unvaccinated	0/3	NP	39.2	NP	+ (≥ 1/32)
Group 1 - #141	Regressive	On-time	0/3	-	39.1	-	+ (1/8)
Group 1 - #251	Regressive	Unvaccinated	0/3	NP	37.6	-	+ (≥ 1/32)
Group 2 - #15	Regressive	Unvaccinated	0/3	-	37.5	-	+ (1/8)
Group 3 - #19	Regressive	Unvaccinated	0/3	-	37.3	-	+ (≥ 1/32)
Group 3 - #5	Regressive	Unvaccinated	0/3	-	37.2	-	+ (≥ 1/32)
Group 1 - #71	Regressive	On-time	0/3	NP	37.1	-	+ (≥ 1/32)
Group 3 - #13	Regressive	Unvaccinated	0/3	-	36.9	-	+ (1/16)
Group 2 - #3	Regressive	Unvaccinated	0/3	-	36.8	-	+ (≥ 1/32)
Group 3 - #7	Regressive	Unvaccinated	0/3	NP	36.6	-	+ (≥ 1/32)
Group 1 - #48	Regressive	Overdue (last given 11.2 years prior)	0/3	-	36.4	-	+ (1/16)
Group 3 - #38	Regressive	Unvaccinated	0/3	-	36.2	39.1	+ (≥ 1/32)
Group 3 - #6	Regressive	Unvaccinated	0/3	-	36.2	-	+ (≥ 1/32)
Group 3 - #36	Regressive	Unvaccinated	0/3	-	35.4	-	+ (≥ 1/32)
Group 3 - #18	Regressive	Unvaccinated	0/3	-	35.3	-	+ (≥ 1/32)
Group 3 - #34	Regressive	Unvaccinated	0/3	-	35.2	-	+ (≥ 1/32)
Group 3 - #17	Regressive	Unvaccinated	0/3	NP	35.1	NP	NP
Group 2 - #22	Regressive	Unvaccinated	0/3	-	35.0	-	+ (≥ 1/32)
Group 1 - #287	Regressive	Unvaccinated	0/3	-	34.9	-	+ (≥ 1/32)
Group 1 - #252	Regressive	Unvaccinated	0/3	-	34.5	-	+ (≥ 1/32)
Group 2 - #38	Regressive	Unvaccinated	0/3	NP	34.4	-	+ (≥ 1/32)
Group 1 - #258	Regressive	Unvaccinated	0/3	-	34.1	-	-
Group 3 - #27	Regressive	Unvaccinated	0/3	-	33.2	-	+ (≥ 1/32)
Group 3 - #15	Regressive	Unvaccinated	0/3	-	33.1	38.4	+ (≥ 1/32)
Group 2 - #13	Regressive	Unvaccinated	0/3	-	32.9	31.8	+ (≥ 1/32)
Group 3 - #33	Regressive	Unvaccinated	0/3	-	31.2	33.7	+ (≥ 1/32)
Group 3 - #52	Regressive	Unvaccinated	0/3	-	31.2	35.9	+ (≥ 1/32)
Group 3 - #29	Regressive	Unvaccinated	0/3	-	31.2	33.7	+ (≥ 1/32)
Group 2 - #36	Regressive	Unvaccinated	0/3	NP	29.4	NP	NP
Group 2 - #4	Regressive	Unvaccinated	0/3	-	28.0	-	+ (≥ 1/32)
**TOTAL POSITIVE** **(presumptively regressive)**		**3/31 vaccinated**	**31 × 0/3**	**0/24**	**31/31**	**6/28**	**27/29**
Group 3 - #3	Progressive	Unvaccinated	1/3 ^1^	+ (43.5%)	37.1	-	+ (≥ 1/32)
Group 3 - #12	Progressive	Unvaccinated	1/3 ^1^	-	36.4	-	+ (≥ 1/32)
Group 1 - #330	Progressive	Unvaccinated	1/3 ^1^	NP	35.3	NP	-
Group 3 - #40	Progressive	Unvaccinated	3/3	+ (13.4%)	31.3	31.1	-
Group 3 - #9	Progressive	Unvaccinated	3/3	+ (6.4%)	31.1	34.1	-
Group 3 - #50	Progressive	Unvaccinated	3/3	- (1.5%)	28.2	27.4	+ (≥ 1/32)
Group 2 - #21	Progressive	Unvaccinated	3/3	+ (24.9%)	22.7	18.9	NP
Group 3 - #51	Progressive	Unvaccinated	3/3	+ (61.2%)	22.5	18.6	-
Group 3 - #10	Progressive	Unvaccinated	3/3	+ (80.0%)	21.5	18.5	-
Group 2 - #30	Progressive	Unvaccinated	3/3	+ (35.1%)	21.4	17.7	NP
Group 3 - #16	Progressive	Unvaccinated	3/3	NP	21.3	NP	-
Group 2 - #31	Progressive	Unvaccinated	3/3	+ (36.6%)	21.3	17.0	NP
Group 2 - #5	Progressive	Unvaccinated	3/3	+ (59.2%)	19.2	17.7	NP
Group 2 - #20	Progressive	Unvaccinated	3/3	+ (27.2%)	19.2	17.1	NP
Group 3 - #26	Progressive	Unvaccinated	3/3	NP	18.4	16.5	-
Group 3 - #31	Progressive	Unvaccinated	3/3	+ (105.2%)	18.3	15.2	-
Group 2 - #7	Progressive	Unvaccinated	3/3	+ (26.7%)	18.2	18.5	NP
Group 2 - #14	Progressive	Unvaccinated	3/3	NP	18.0	NP	-
Group 3 - #39	Progressive	Unvaccinated	3/3	+ (49.3%)	17.8	15.6	-
Group 3 - #42	Progressive	Unvaccinated	3/3	+ (65.2%)	17.6	15.3	-
Group 1 - #62	Progressive	On-time	3/3	+ (55.9%)	16.8	18.7	-
**TOTAL POSITIVE** **(presumptively progressive)**		**1/21 vaccinated**	**3 × 1/3, 18 × 3/3**	**15/17**	**21/21**	**16/18**	**3/15**

^1^ only tested p27-positive with SNAP Combo^®^.

**Table 5:** Outcomes of FeLV-infected cats in Groups 1 and 2 at the time of writing. All were non-pedigree cats except for #141 in Group 1 that was a Tonkinese, #258 in Group 1 that was a Chinchilla and #4 in Group 2 that was a Birman. Due to the field nature of the study, it is not known how long cats had been infected for at the time of diagnosis. When known, the time elapsed between diagnosis and final outcome is noted. The approximate time between diagnosis and manuscript writing was 4–6 years. MN = male neutered, FS = female spayed, CKD = chronic kidney disease.

Cat ID	Age at Time of Diagnosis (Years)	Sex	Outcome
**PRESUMPTIVELY REGRESSIVE INFECTIONS**			
Group 1 - #41	15	MN	Found dead 25 months after diagnosis, suspected due to progression of CKD
Group 1 - #48	12	MN	Lost to follow up
Group 1 - #71	6	FS	Alive and doing well at the time of writing
Group 1 - #141	15.8	MN	Euthanased 15 months after diagnosis due to progression of CKD
Group 1 - #251	2.3	MN	Lost to follow up
Group 1 - #252	4.9	MN	Lost to follow up
Group 1 - #258	10.2	FS	Lost to follow up
Group 1 - #287	7.6	MN	Lost to follow up
Group 1 - #386	11.4	MN	Lost to follow up
Group 2 - #3	10	FS	Alive and doing well at the time of writing. Stable diabetic (in remission at the time of writing)
Group 2 - #4	14	FS	Euthanased due to suspected cardiomyopathy
Group 2 - #13	10	MN	Euthanased due to geriatric-related disease
Group 2 - #15	5	FS	Alive and doing well at the time of writing
Group 2 - #22	4	MN	Found dead, suspected due to brown snake bite
Group 2 - #36	13	FS	Euthanased due to progression of CKD
Group 2 - #38	3	FS	Lost to follow up (ran away)
**PRESUMPTIVELY PROGRESSIVE INFECTIONS**			
Group 1 - #62	3.4	MN	Euthanased 8 months after diagnosis since not doing well (inappetent and constipated)
Group 1 - #330	6.4	FS	Alive and doing well at the time of writing
Group 2 - #5	11	FS	Euthanased 24 months after diagnosis due to mass in abdomen, post-mortem not performed
Group 2 - #7	11	FS	Euthanased due to progression of CKD
**Group 2 - #14**	4	MN	Euthanased due to multiple diffuse skin masses, one month after FeLV diagnosis. Feline sarcoma virus (FeSV) infection was diagnosed post-mortem (histopathology and gp70 staining of masses)
**Group 2 - #20**	8	MN	Euthanased 38 months after diagnosis due to nasal cavity tumour
**Group 2 - #21**	9	MN	Euthanased 13 months after diagnosis due to breathing difficulties, cardiac disease suspected due to severe heart murmur, post-mortem not performed, co-infected with FIV
**Group 2 - #30**	2	MN	Euthanased 22 months after diagnosis due to breathing difficulties, post-mortem revealed mediastinal lymphoma
**Group 2 - #31**	2	MN	Euthanased 6 months after diagnosis, severe non-regenerative anaemia and icterus, post-mortem revealed hepatomegaly, splenomegaly, pericardial effusion and pleural effusion

## References

[1] Levy J., Crawford C., Hartmann K., Hofmann-Lehmann R., Little S., Sundahl E., Thayer V. (2008). American Association of Feline Practitioners’ feline retrovirus management guidelines. J. Feline Med. Surg..

[2] Lutz H., Addie D., Belák S., Boucraut-Baralon C., Egberink H., Frymus T., Gruffydd-Jones T., Hartmann K., Hosie M.J., Lloret A. (2009). Horzinek, Feline Leukaemia: ABCD Guidelines on Prevention and Management. J. Feline Med. Surg..

[3] Firth C.L., Möstl K. (2015). A survey of feline leukaemia virus antigenaemia among cats in eastern Austria: A retrospective analysis of serum samples routinely tested between 1996 and 2011. J. Feline Med. Surg. Open Rep..

[4] Ravi M., Wobeser G.A., Taylor S.M., Jackson M.L. (2010). Naturally acquired feline immunodeficiency virus (FIV) infection in cats from western Canada: Prevalence, disease associations, and survival analysis. Can. Vet. J..

[5] Westman M.E., Paul A., Malik R., McDonagh P., Ward M.P., Hall E., Norris J.M. (2016). Seroprevalence of feline immunodeficiency virus and feline leukaemia virus in Australia: Risk factors for infection and geographical influences (2011–2013). J. Feline Med. Surg. Open Rep..

[6] Hofmann-Lehmann R., Gonczil E., Riond B., Meli M.L., Willi B., Howard J., Schaarschmidt D., Regli W., Gilli U., Boretti F.S. (2018). Feline leukemia virus infection: Importance and current situation in Switzerland. Schweizer Archiv. Fur. Tierheilkunde.

[7] Willett B.J., Hosie M.J. (2013). Feline leukaemia virus: Half a century since its discovery. Vet. J..

[8] Möstl K., Egberink H., Addie D., Frymus T., Boucraut-Baralon C., Truyen U., Hartmann K., Lutz H., Gruffydd-Jones T., Radford A.D. (2013). Prevention of infectious diseases in cat shelters: ABCD guidelines. J. Feline Med. Surg..

[9] de Almeida N.R., Danelli M.G., da Silva L.H., Hagiwara M.K., Mazur C. (2012). Prevalence of feline leukemia virus infection in domestic cats in Rio de Janeiro. J. Feline Med. Surg..

[10] Biezus G., Machado G., Ferian P.E., da Costa U.M., Pereira L.H.H.d.S., Withoeft J.A., Nunes I.A.C., Muller T.R., de Cristo T.G., Casagrande R.A. (2019). Prevalence of and factors associated with feline leukemia virus (FeLV) and feline immunodeficiency virus (FIV) in cats of the state of Santa Catarina, Brazil. Comp. Immun. Microbiol. Infect. Dis..

[11] Bande F., Arshad S.S., Hassan L., Zakaria Z., Sapian N.A., Rahman N.A., Alazawy A. (2012). Prevalence and risk factors of feline leukaemia virus and feline immunodeficiency virus in peninsular Malaysia. BMC Vet. Res..

[12] Chan C.T., Beatty J.A., Barrs V.R., Morris A.K., Reagan K., Lappin M.R. (2013). Evidence for feline immunodeficiency virus, feline leukemia virus, Bartonella spp, haemoplasmas, and Toxoplasma spp. exposure in Singaporean cats. J. Vet. Intern. Med..

[13] Sukhumavasi W., Bellosa M.L., Lucio-Forster A., Liotta J.L., Lee A.C.Y., Pornmingmas P., Chungpivat S., Mohammed H.O., Lorentzen L., Dubey J.P., Bowman D.D. (2012). Serological survey of Toxoplasma gondii, Dirofilaria immitis, Feline Immunodeficiency Virus (FIV) and Feline Leukemia Virus (FeLV) infections in pet cats in Bangkok and vicinities, Thailand. Vet. Parasitol..

[14] Westman M.E., Malik R., Hall E., Sheehy P.A., Norris J.M. (2017). Comparison of three feline leukaemia virus (FeLV) point-of-care antigen test kits using blood and saliva. Comp. Immun. Microbiol. Infect. Dis..

[15] Hardy W.D., Hess P.W., MacEwen E.G., McClelland A.J., Zuckerman E.E., Essex M., Cotter S.M., Jarrett O. (1976). Biology of feline leukemia virus in the natural environment. Cancer Res..

[16] Hartmann K. (2012). Clinical aspects of feline retroviruses: A review. Viruses.

[17] Hofmann-Lehmann R., Cattori V., Tandon R., Boretti F.S., Meli M.L., Riond B., Pepin A.C., Willi B., Ossent P., Lutz H. (2007). Vaccination against the feline leukaemia virus: Outcome and response categories and long-term follow-up. Vaccine.

[18] Englert T., Lutz H., Sauter-Louis C., Hartmann K. (2012). Survey of the feline leukemia virus infection status of cats in Southern Germany. J. Feline Med. Surg..

[19] Cristo T.G., Biezus G., Noronha L.F., Pereira L.H.H.S., Withoeft J.A., Furlan L.V., Costa L.S., Traverso S.D., Casagrande R.A. (2019). Feline lymphoma and a high correlation with feline leukaemia virus infection in Brazil. J. Comp. Pathol..

[20] Helfer-Hungerbuehler A.K., Widmer S., Kessler Y., Riond B., Boretti F.S., Grest P., Lutz H., Hofmann-Lehmann R. (2015). Long-term follow up of feline leukemia virus infection and characterization of viral RNA loads using molecular methods in tissues of cats with different infection outcomes. Virus Res..

[21] Gabor L.J., Jackson M.L., Trask B., Malik R., Canfield P.J. (2001). Feline leukaemia virus status of Australian cats with lymphosarcoma. Aust. Vet. J..

[22] Jackson M.L., Haines D.M., Meric S.M., Misra V. (1993). Feline leukemia virus detection by immunohistochemistry and polymerase chain reaction in formalin-fixed, paraffin-embedded tumor tissue from cats with lymphosarcoma. Can. J. Vet. Res..

[23] McLuckie A., Barrs V., Lindsay S., Aghazadeh M., Sangster C., Beatty J. (2018). Molecular Diagnosis of Felis catus Gammaherpesvirus 1 (FcaGHV1) Infection in Cats of Known Retrovirus Status with and without Lymphoma. Viruses.

[24] Westman M.E., Malik R., Hall E., Harris M., Norris J.M. (2016). The protective rate of the feline immunodeficiency virus vaccine: An Australian field study. Vaccine.

[25] Westman M.E., Malik R., Hall E., Sheehy P.A., Norris J.M. (2015). Determining the feline immunodeficiency virus (FIV) status of FIV-vaccinated cats using point-of-care antibody kits. Comp. Immun. Microbiol. Infect. Dis..

[26] Jarrett O., Ganiere J.P. (1996). Comparative studies of the efficacy of a recombinant feline leukaemia virus vaccine. Vet. Rec..

[27] Hofmann-Lehmann R., Tandon R., Boretti F.S., Meli M.L., Willi B., Cattori V., Gomes-Keller M.A., Ossent P., Golder M.C., Flynn J.N., Lutz H. (2006). Reassessment of feline leukaemia virus (FeLV) vaccines with novel sensitive molecular assays. Vaccine.

[28] Torres A.N., O’Halloran K.P., Larson L.J., Schultz R.D., Hoover E.A. (2010). Feline leukemia virus immunity induced by whole inactivated virus vaccination. Vet. Immunol. Immunopathol..

[29] Hofmann-Lehmann R., Cattori V., Tandon R., Boretti F.S., Meli M.L., Riond B., Lutz H. (2008). How molecular methods change our views of FeLV infection and vaccination. Vet. Immunol. Immunopathol..

[30] Westman M.E., Malik R., Norris J.M. (2019). Diagnosing feline immunodeficiency virus (FIV) and feline leukaemia virus (FeLV) infection: An update for clinicians. Aust. Vet. J..

[31] Tandon R., Cattori V., Gomes-Keller M.A., Meli M.L., Golder M.C., Lutz H., Hofmann-Lehmann R. (2005). Quantitation of feline leukaemia virus viral and proviral loads by TaqMan (R) real-time polymerase chain reaction. J. Virol. Methods.

[32] Helps C.R., Lait P., Damhuis A., Björnehammar U., Bolta D., Brovida C., Chabanne L., Egberink H., Ferrand G., Fontbonne A. (2005). Factors associated with upper respiratory tract disease caused by feline herpesvirus, feline calicivirus, Chlamydophila felis and Bordetella bronchiseptica in cats: Experience from 218 European catteries. Vet. Rec..

[33] Pinches M.D., Helps C.R., Gruffydd-Jones T.J., Egan K., Jarrett O., Tasker S. (2007). Diagnosis of feline leukaemia virus infection by semi-quantitative real-time polymerase chain reaction. J. Feline Med. Surg..

[34] Lutz H., Pedersen N.C., Durbin R., Theilen G.H. (1983). Monoclonal antibodies to three epitopic regions of feline leukemia virus p27 and their use in enzyme-linked immunosorbent assay of p27. J. Immunol. Methods.

[35] Hofmann-Lehmann R., Huder J.B., Gruber S., Boretti F., Sigrist B., Lutz H. (2001). Feline leukaemia provirus load during the course of experimental infection and in naturally infected cats. J. Gen. Virol..

[36] Monograph of the European Pharmacopoeia Commission (2005). Council of Europe, and Convention on the Elaboration of a European Pharmacopoeia. Feline leukemia vaccine (inactivated). European Pharmacopoiea.

[37] Beatty J.A., Tasker S., Jarrett O., Lam A., Gibson S., Noe-Nordberg A., Phillips A., Fawcett A., Barrs V.R. (2011). Markers of feline leukaemia virus infection or exposure in cats from a region of low seroprevalence. J. Feline Med. Surg..

[38] Gomes-Keller M.A., Gönczi E., Tandon R., Riondato F., Hofmann-Lehmann R., Meli M.L., Lutz H. (2006). Detection of feline leukemia virus RNA in saliva from naturally infected cats and correlation of PCR results with those of current diagnostic methods. J. Clin. Microbiol..

[39] Malik R., Kendall K., Cridland J., Coulston S., Stuart A.J., Snow D., Love D.N. (1997). Prevalences of feline leukaemia virus and feline immunodeficiency virus infections in cats in Sydney. Aust. Vet. J..

[40] Norris J.M., Bell E.T., Hales L., Toribio J.A.L., White J.D., Wigney D.I., Baral R.M., Malik R. (2007). Prevalence of feline immunodeficiency virus infection in domesticated and feral cats in eastern Australia. J. Feline Med. Surg..

[41] Bęczkowski P.M., Litster A., Lin T.L., Mellor D.J., Willett B.J., Hosie M.J. (2015). Contrasting clinical outcomes in two cohorts of cats naturally infected with feline immunodeficiency virus (FIV). Vet. Microbiol..

[42] Wilson S., Greenslade J., Saunders G., Holcroft C., Bruce L., Scobey A., Childers T., Sture G., Thompson J. (2012). Difficulties in demonstrating long term immunity in FeLV vaccinated cats due to increasing age-related resistance to infection. BMC Vet. Res..

[43] Fischer A., Benka V.A., Briggs J.R., Maki J., Morris K.N., Myers K.A., Rhodes L., Weedon G.R., Levy J.K. (2018). Hybrid model intermediate between a laboratory and field study: A humane paradigm shift in feline research. J. Feline Med. Surg..

[44] Boenzli E., Hadorn M., Hartnack S., Huder J., Hofmann-Lehmann R., Lutz H. (2014). Detection of antibodies to the feline leukemia virus (FeLV) transmembrane protein p15E: an alternative approach for serological FeLV detection based on antibodies to p15E. J. Clin. Microbiol..

[45] Hartmann K., Day M.J., Thiry E., Lloret A., Frymus T., Addie D., Boucraut-Baralon C., Egberink H., Gruffydd-Jones T., Horzinek M.C., Hosie M.J. (2015). Feline injection site sarcoma: ABCD guidelines on prevention and management. J. Feline Med. Surg..

[46] Sparkes A.H. (2003). Feline leukaemia virus and vaccination. J. Feline Med. Surg..

[47] Torres A.N., Mathiason C.K., Hoover E.A. (2005). Re-examination of feline leukemia virus: Host relationships using real-time PCR. Virology.

[48] Patel M., Carritt K., Lane J., Jayappa H., Stahl M., Bourgeois M. (2015). Comparative Efficacy of Feline Leukemia Virus (FeLV) Inactivated Whole-Virus Vaccine and Canarypox Virus-Vectored Vaccine during Virulent FeLV Challenge and Immunosuppression. Clin. Vaccine Immunol..

[49] Stuke K., King V., Southwick K., Stoeva M.I., Thomas A., Winkler M.T.C. (2014). Efficacy of an inactivated FeLV vaccine compared to a recombinant FeLV vaccine in minimum age cats following virulent FeLV challenge. Vaccine.

[50] Rojko J.L., Hoover E.A., Quackenbush S.L., Olsen R.G. (1982). Reactivation of latent feline leukaemia virus infection. Nature.

[51] Nesina S., Helfer-Hungerbuehler A.K., Riond B., Boretti F.S., Willi B., Meli M.L., Grest P., Hofmann-Lehmann R. (2015). Retroviral DNA - the silent winner: blood transfusion containing latent feline leukemia provirus causes infection and disease in naive recipient cats. Retrovirology.

[52] Pennisi M.G., Hartmann K., Addie D.D., Lutz H., Gruffydd-Jones T., Boucraut-Baralon C., Egberink H., Frymus T., Horzinek M.C., Hosie M.J. (2015). Blood transfusion in cats: ABCD guidelines for minimising risks of infectious iatrogenic complications. J. Feline Med. Surg..

[53] Jarrett O., Pacitti A.M., Hosie M.J., Reid G. (1991). Comparison of diagnostic methods for feline leukemia virus and feline immunodeficiency virus. J. Am. Vet. Med. Assoc..

[54] Miyazawa T., Jarrett O. (1997). Feline leukaemia virus proviral DNA detected by polymerase chain reaction in antigenaemic but non-viraemic (‘discordant’) cats. Arch. Virol..

